# What is needed by parents of constipated infants and toddlers: A cross-sectional study in China

**DOI:** 10.3389/fped.2023.1066355

**Published:** 2023-04-12

**Authors:** Yuanyuan Wang, Jinjin Cao, Weiying Zhang, Hongyu Chen, Mei Li, Zhifeng Liu, Jianan Wang

**Affiliations:** ^1^ Department of Nursing, Children's Hospital of Nanjing Medical University, Nanjing, China; ^2^ Department of Gastroenterology, Children's Hospital of Nanjing Medical University, Nanjing, China; ^3^ Department of Emergency, Children's Hospital of Nanjing Medical University, Nanjing, China; ^4^ Department of Neurosurgery, The First Hospital of China Medical University, Shenyang, China

**Keywords:** functional constipation, infant, need assessment, caregiving, parents

## Abstract

**Background:**

Childhood functional constipation is a worldwide problem that affects the intestinal function of children and the quality of life of their families. Treatment and management of the disease need to be carried out at home by parents. Assessment of caregiving needs is an important link in planning and implementing the intervention. This study aimed to assess the caregiving needs of parents of FC infants and toddlers.

**Methods:**

The researchers recruited convenience samples of parents from an outpatient pediatric constipation clinic of a children's medical center. Totally 211 fathers/mothers were recruited. Nursing needs were measured by a questionnaire, and associations between nursing needs and potential factors were examined using multiple regression analysis.

**Results:**

The vast majority of participants (88.7%) expressed the need of receiving support from professionals, and only 44 (20.85%) had obtained help from medical staff. The needs of parents mainly include information needs, health needs, psychological needs, and social needs. Of all the needs, the highest score was for information needs (3.87 ± 0.69), followed by the dimension of health needs (3.74 ± 0.82). Results showed statistically significant differences in parental education, place of residence, age of children, duration of FC, defecation frequency, difficulty of defecation, and stool traits in nursing needs (*p *< 0.05). The regression model explained 64.2% of the variance of nursing needs.

**Conclusions:**

Information needs were the major concern for parents, and the unmet needs of parents should be addressed during treatment and care. When developing care plans and providing health education, it should be adjusted according to the specific conditions of the child and parents to improve the compliance of the parents with treatment and care.

## 1. Introduction

Functional constipation (FC) is defined as constipation without an organic etiology and is diagnosed according to the Rome criteria (1). It is one of the most frequent reasons for visits to pediatric clinics of all ages, accounting for 95% of constipation in children (2, 3). In a recent review by Koppen (4), the pooled prevalence of childhood FC was 9.5%, with no gender differences.

Except for the high prevalence, FC poses a significant burden on health budgets, a report from England showed that FC cost the English National Health Service (NHS) £168 million in 2020–21 (5). Compared with other globally common, frustrating, and long-lasting disorders such as childhood asthma and migraine, children with constipation require seven times as much medical attention as asthma and three times as much as migraines (6, 7). According to research, functional constipation persists into adulthood and becomes a chronic condition in about one-third of affected children (8), thus having a continuous impact on health-related quality of life (9), and leading to a series of behavioral and emotional problems (10, 11). The etiology and pathophysiology of functional constipation are multi-factorial, and all causes are not mutually exclusive and may exist simultaneously. The initial symptoms are often painful defecation which leads to stool withholding. When more water was absorbed from the retained stool, the rectal sensation will be weakened and the child will lose the normal defecation impulse, which is further affecting gastrointestinal function (12, 13). Besides, Children with functional constipation often have a family history of constipation, and parental factors such as socioeconomic level, educational level, and parental rearing attitudes are closely related to childhood FC. In addition, toilet training issues, dietary causes, and stressful life events such as psychological or physical trauma are also important causes of FC (14).

To date, the recommended standard treatment for childhood FC includes diet therapy, constipation family education, toilet training, drug use, and behavior change (8). These treatments often need to be done at home by parents. The disease and the struggle to treat it at home can be challenging for parents (15, 16). The complexity of disease etiology and the burdensome and long-term treatment make parents feel overwhelmed, and various needs exist from diagnosis to the end of treatment. To provide an appropriate education for parents, it is necessary to determine what needs exist and the corresponding level of needs. Focusing on parents' care needs is an important first step in providing them with matched professional guidance, which is essential for successful disease management. Previous study have shown that gaps exist between the strategies offered by healthcare professionals and the caregiving needs of parents leading to poor communication and understanding gaps between them (17). If the health guidance cannot cover the caregiving needs, the incorrect behavior of parents harms the benign outcome of the disease. For example, previous studies have shown that failure to conduct defecation training in time or wrong defecation training are risk factors for FC (18). Therefore, it is necessary to understand caregiving needs to provide continuous and scientific care for children, maintain the physical and mental health of the main caregivers, reduce the psychological burden, and improve the clinical outcome of children (19). In general, childhood constipation tends to be rooted in infancy and early childhood. Therefore, it is very important to pay attention to the needs of parents of infants and toddlers and to provide help as early as possible. Previous studies have mostly used qualitative methods to explore parental needs (15, 20, 21), however, compared with qualitative research, quantitative research can not only investigate the content of parents' needs but also clarify the degree of various needs, so that our interventions can be more targeted.

In summary, our purpose was to investigate the caregiving needs of parents of FC infants and toddlers using a quantitative study. Besides, influencing factors were analyzed to better understand parental needs, promote effective communication between medical staff and parents, and ensure the effectiveness of health education implementation.

## 2. Methods

### 2.1. Design

A quantitative, cross-sectional, questionnaire-based design was used in this study to (a) investigate the caregiving needs of parents of infants and toddlers with FC and (b) the influencing factors of their needs. The study was conducted and reported according to the Strengthening the Reporting of Observational Studies in Epidemiology (STROBE) checklist (Supplementary File S1).

### 2.2. Participants and settings

Parents of infants and toddlers who visited the outpatient department of the Children's Medical Center of Jiangsu Province were recruited. Convenience sampling was performed, with the following criteria: inclusion criteria for children (a) Were of Chinese nationality; (b) Age ranged from 28 days to 3 years old; (c) Met the Rome IV criteria of FC; Inclusion criteria for parents: (a) Were able to speak Mandarin, understood the contents of the questionnaire, and clearly expressed personal opinions; (b) Undertook the main task of taking care of the children, and took care of the children for the longest time per day; (c) Aged between 18 and 55; (d) Were willing to participate and signed consent.

### 2.3. Measures

#### 2.3.1. General information

Self-compiled, including two parts: (1) general data of children, including sex, age, defecation interval, defecation time and stool trait, constipation duration, and medical payment methods. Stool traits were classified according to the Bristol stool scale, type I = separate hard lumps, type II = lumpy and sausage-like, type III = a sausage shape with cracks in the surface, type IV = like a smooth, soft sausage or snake, type V = soft blobs with clear-cut edges, type VI = mushy consistency with ragged edges, type VII = liquid consistency with no solid pieces; (2) general data of parents, including age, education, place of residence, occupation, family average monthly income.

#### 2.3.2. Development of the questionnaire

The development of the Caregiving needs questionnaire of parents of FC children mainly comprised two stages:

Stage 1: The construction of core components of the questionnaire

Firstly, we built the conceptual framework and dimensions of the scale based on the literature review. We searched the following databases of Web of Science, PubMed databases, China National Knowledge Infrastructure (CNKI), Wanfang Database, and VIP database with the keywords “caregiving needs” “nursing needs” “infant” “children” “parents” “caregiver” from the establishment of the database to May 2021. In addition, identified other publications by manually searching references in included publications. Finally, we divided the caregiving needs of parents into dimensions and items from four aspects through literature review and relevant scale (20–24): information needs, health needs, psychological needs, and social needs. Besides, we have set up a WeChat follow-up group of parents in June 2021 to collect the problems and needs of the parents in the process of caring for FC children. We collect information mainly through the following questions in the WeChat group: ① Can you talk about the needs and feelings of caring for a constipated child? ② What are the physical and psychological changes involved in caring for a constipated child? ③ What support or assistance you would like to receive in caring for a constipated child? The contents were analyzed and extracted according to the results, the initial items of the scale constructed in the previous stage were adjusted and supplemented, and a 35-item questionnaire was developed.

Stage 2: Questionnaire development.

Based on the 35-item questionnaire, an expert group meeting was conducted. Ten gastroenterologists and clinical nursing specialists were invited to the meeting (all with bachelor's degree or above with ten year working period). The person in charge of this project chaired the meeting and invited experts to read the content of the questionnaire. The experts put forward suggestions and modifications to the specific content, scientificity, and feasibility of the questionnaire. The researcher modifies the questionnaire according to the experts' opinions and reviewed it again with all experts to form the final version of the questionnaire. After the meeting, the researcher collects the experts' information questionnaire in time. After redundant components were eliminated, a 4-dimensional 19-item questionnaire was formed (Table 1). The questions included 5-point Likert-type questions (1 for not needed at all, 5 for very much needed) The higher the score, the higher the caregiving needs of the parents.

**Table 1:** Caregiving needs questionnaire of parents of FC children.

Knowledge needs	1: Not needed at all	2: Not needed	3: Indifferent	4: Needed	5: Very much needed
I need an introduction to the causes of functional constipation					
I need the introduction of auxiliary tests and guidance on how to cooperate with the tests					
I need guidance on self-assessment for constipation					
I need medication guidance for functional constipation					
I need to know about complications and guidance on preventive measures					
I need dietary and nutritional guidance					
**Health Needs**					
I need guidance on how to deal with bloating					
I need guidance on how to deal with hard stools					
I need guidance on how to deal with defecation difficulties					
I need to know how to do bowel training					
I need guidance on the methods of promoting intestinal movement					
**Psychological Needs**					
I need to know how to relieve the anxiety caused by my child's illness					
I need guidance on ways to get support from my family					
I need guidance on how to talk to people close to me about my feelings					
I need to know how to relieve the guilt caused by my child's illness					
**Social Needs**					
I would like to attend lectures by leading experts					
I need health consultation services in the form of phone calls, text messages, etc.					
I would like to get help to attend constipation-related events and share information with other families					
I would like help in keeping in touch with other family members (phone/WeChat, etc.)					

In August 2021, a preliminary survey was conducted to assess surface validity among the parents of 30 FC children who met the inclusion criteria. Parents were invited to make suggestions on the time required to fill in the questionnaire, legibility, and relevant content. The time to complete the questionnaire was 5–10 min. The content validity of the scale was evaluated by expert consultation. Six experts (2 clinical nursing experts, 2 nursing research experts, and 2 senior gastroenterologists, all with bachelor's degree or above with ten year working period) were selected to evaluate the questionnaire. The item level content validity (I-CVI) of the questionnaire was 0.83–1.00, and S-CVI/Ave was 0.98 > 0.90, indicating that the content validity was good. The Cronbach's *α* coefficients of the four parts were 0.747, 0.738, 0.744, and 0.793, and the Cronbach's *α* coefficient of the total questionnaire was 0.947, indicating good reliability.

### 2.4. Data analysis

According to Kendall's (25) experience and method of multivariate linear regression sample content estimation, the sample size should be 10–20 times the number of independent variables. There were 19 independent variables in this study, combined with the effective recovery rate of a presurvey questionnaire, and considering 10% sample loss, the sample size was finally determined to be 237 cases.

Entered the raw data into Microsoft Excel (2010 version) and SPSS 26.0, and double-checked the accuracy of the data. After screening for eligibility and completeness of responses as described earlier, the numerical variables’ normality distribution was established using descriptive statistics, graphic analysis, and the P–P test. Quantitative data were described with means, standard deviations, medians, and ranges; frequencies and percentages were used to describe categorical variables. Independent-sample *t*-tests or one-way analysis of variance were used to explore the differences between groups. All statistical analyses were performed with SPSS. Statistical significance was defined as a *p *< 0.05.

## 3. Results

### 3.1. Description of the samples

Two hundred and thirty-seven participants were invited to participate and 235 responded. After additional screening for the usability of data described earlier, responses from 211 participants were included in the final analysis, yielding a response rate of 89.03%. Their demographic characteristics were summarized in Table 2. Among 211 families, 150 (71.09%) had others to help take care of their children, helpers ranging from one to four. There were 142 families with only one child, while the other 69 had two or more children.

**Table 2:** Demographic information and single-factor analysis in nursing needs (*N* = 211).

Category			Mean (SD) or *N* (%)	Total needs score	*t*/*F*	*p*-value
**Parents**						
Age (year)	Mother		27.8 (3.5)		0.001	0.970
	Father		28.2 (3.7)		0.052	0.820
Education	Mother	Middle school	8 (3.8)	58.13 ± 9.92	17.382	**0**.**000**
		High school	34 (16.1)	58.94 ± 10.47		
		College	125 (59.2)	69.46 ± 8.47		
		Above graduate school	44 (20.9)	71.39 ± 9.17		
	Father	Middle school	2 (0.9)	58.50 ± 6.50	3.048	**0**.**030**
		High school	39 (18.5)	65.15 ± 10.20		
		College	120 (56.9)	69.43 ± 8.77		
		Above graduate	50 (23.7)	66.08 ± 12.01		
Occupation	Mother	Institutions	50 (23.7)	68.30 ± 9.59	1.908	0.110
		company	94 (44.5)	69.11 ± 9.84		
		workers	10 (4.7)	66.80 ± 9.95		
		farmers	27 (12.8)	67.07 ± 10.37		
		freelance	30 (14.2)	63.43 ± 10.26		
	Father	Institutions	42 (19.9)	68.81 ± 9.33	0.765	0.549
		Company	131 (62.1)	68.11 ± 10.08		
		Workers	22 (10.4)	65.91 ± 9.19		
		Farmers	2 (0.9)	65.00 ± 3.00		
		Freelance	14 (6.6)	64.36 ± 12.96		
Monthly income (RMB)	≤5,000		4 (1.9)	62.00 ± 12.19	2.062	0.106
	5,001–10,000		39 (18.5)	64.67 ± 11.44		
	10,001–15,000		94 (44.5)	68.70 ± 9.58		
	>15,000		74 (35.1)	68.45 ± 9.42		
Helper	Yes		150 (71.1)	67.42 ± 10.53	0.515	0.474
	No		61 (28.9)	68.52 ± 8.90		
Number of children	1		135 (67.3)	68.36 ± 12.10	1.392	0.239
	2		68 (32.2)	67.65 ± 9.45		
	3		8 (0.5)	58.13 ± 9.83		
Living place	Urban		113 (53.6)	72.19 ± 7.64	7.779	**0**.**001**
	Rural		98 (46.4)	62.60 ± 10.14		
**Children**						
Gender	Boys		126 (59.7)	67.32 ± 9.72	−0.736	0.462
	Girls		85 (50.2)	68.36 ± 10.60		
Age	0–11 months		32 (40.3)	57.75 ± 8.09	0.466	**0**.**000**
	1–3 years		179 (84.8)	69.53 ± 9.35		
Defecation interval	>2 times/week		6 (2.8)	53.33 ± 6.80	29.104	**0**.**000**
	4–5 days		14 (6.6)	53.29 ± 7.75		
	6–7 days		85 (40.3)	65.85 ± 8.61		
	>7 days		106 (50.2)	71.98 ± 8.53		
Defecation difficulty	None		3 (1.4)	65.67 ± 10.78	42.475	**0**.**000**
	Mild		12 (5.7)	57.00 ± 8.40		
	Moderate		80 (37.9)	63.89 ± 9.11		
	Severe		116 (55.0)	71.56 ± 9.00		
Defecation time	<10 min		63 (29.9)	65.51 ± 11.16	2.165	0.093
	10–15 min		66 (31.3)	67.44 ± 8.57		
	16–20 min		45 (21.3)	70.16 ± 8.92		
	> 20 min		37 (17.5)	72.88 ± 10.13		
Abdominal distension	None		118 (55.9)	67.53 ± 9.10	1.687	0.171
	Occasionally		41 (19.4)	69.27 ± 10.59		
	Sometimes		41 (19.4)	65.61 ± 11.55		
	Often		11(5.2)	72.27 ± 10.23		

Bold values: *p*<0.05.

### 3.2. Results of caregiving needs

For 211 groups of parents, the vast majority (*n* = 187, 88.7%) expressed need or great need of help from the professionals, and a small number (*n* = 24, 11.3%) indicated that they don't care or don't want help. Almost all parents said they had received help from family members previously, while 112 groups (53.08%) had received help from friends, colleagues, or neighbors, and only 44 (20.85%) said they had received help from medical staff. Analysis *via* the p–p indicated the total score and scores for each item were close to normally distributed, so we chose parameter test to analyze the data. The average total score for caregiving needs was 67.86 ± 9.96 (range, 19–95 points). Generally, parents had a moderate level of needs.

As shown in Table 3, the dimension with the highest score was information needs with 3.87 (SD: 0.69), followed by the dimension of health needs with 3.74 (SD: 0.82), and the lowest score is the dimension of psychological needs with 3.15 (SD: 0.87). The five items with the highest score were mainly distributed in the dimension of information needs and health needs. The information needs included constipation cause introduction, self-assessment method guidance, and diet and nutrition guidance, and health needs included defecation difficulty management and intestinal movement promotion guidance, with an average score from 4.01 to 4.43 (Table 4).

**Table 3:** Mean and standard deviations of variables of nursing needs (*N* = 211).

Variables	M	SD	Min.	Max.	Score rate
Information needs	3.87	0.69	2.00	5.00	77.50%
Health needs	3.74	0.82	1.40	5.00	74.73%
Psychological needs	3.15	0.87	1.00	5.00	62.94%
Social needs	3.30	0.83	1.00	5.00	66.09%
Total score	67.74	10.12	32	88	71.30%

*M, mean; SD, standard deviation; Min, minimum score; Max, maxmum score.

**Table 4:** The five items with the highest scores.

No.	Item	Mean ± SD	Score rate
1	Nursing care of defecation difficulties	4.43 ± 0.88	88.63%
2	An introduction to the causes of functional constipation	4.26 ± 0.93	85.12%
3	Diet and nutrition guidance	4.18 ± 0.99	83.60%
4	Guidance on the methods of promoting intestinal movement	4.04 ± 1.07	80.85%
5	Guidance of self-assessment	4.01 ± 0.94	80.19%

### 3.3. Factors associated with needs

The linear regression results among nursing needs and socio-demographic variables.

Demographic and disease-related data were used as independent variables, and questionnaire scores were used as dependent variables, independent sample *T*-test, and one-way ANOVA. The results showed that there were statistically significant differences in parental education, place of residence, age of children, duration, frequency, the difficulty of defecation, and fecal traits in nursing needs (*p* < 0.05). LSD multiple tests was used to further pally compare the multiple classification variables.

It was found that the mothers with college or undergraduate education, postgraduate education or above had higher scores than those with high school education or below (*p* < 0.05). Those fathers who had a college education or undergraduate education had higher scores than those who had high school or technical secondary education (*p* = 0.021), and higher than fathers with graduate education or above (*p* = 0.048). The scores of parental nursing needs of children with defecation intervals of 6–7 days were higher than those with 4–5 days per time (*p* = 0.000). At the same time, it was higher than those over 2 times/week (*p* = 0.004), and those beyond 7 days had the highest score of parental nursing needs, and the difference was statistically significant compared with other groups (*p* < 0.05). In terms of defecation difficulty, the score of severe defecation difficulty was the highest, and the difference was statistically significant (*p* < 0.05). Meanwhile, the score of moderate defecation difficulty was higher than that of mild (*p* < 0.05). AS for stool trait, parents of children with type I had the highest score, there was statistically significant compared with other groups (*p* < 0.001), and scores of type II were higher than those of type III, the difference was statistically significant (*p* < 0.001), but there was no statistically significant between type II and IV.

When analyzed by parental nursing needs score as the dependent variable, parental education level, place of residence, age of children, defecation interval, defecation difficulty, and stool traits were statistically significant (*p *< 0.05), by contrast, no significant differences were found in the age of parents, occupation, monthly income, helper, number of children, gender of children, defecation time, abdominal distension (*p *> 0.05). Taking total score as the dependent variable, area, parental education level, place of residence, age of children, defecation interval, defecation difficulty, and stool traits as an independent variable. Dummy variables were set for the above variables, with education status as middle school, defecation interval >2 times/week, stool trait as type IV or above, and no defecation difficulty as the reference variables (Table 5). The results showed that residence and stool traits were the main influencing factors for care needs (Table 6).

**Table 5:** Independent variable assignment methods.

Independent variable	Methods
Education of mothers	Middle school = 0, High school = 1, College = 2, Above graduate school = 3
Education of fathers	Middle school = 0, High school = 1, College = 2, Above graduate school = 3
Living place	Rural = 0, Urban = 1
Age	0–11 months = 0, 1–3 years = 1
Defecation interval	>2 times/week = 0, 4–5 days = 1, 6–7 days = 2, >7 days = 3
Stool trait	Type IV or above = 0, Type III = 1, Type II = 2, type I = 3
Defecation difficulty	None = 0, Mild = 1, Moderate = 2, Severe = 3

**Table 6:** Regression analysis of multiple factors in nursing needs (*N* = 211).

Dependent variable	Regression coefficient	SE	Standardized regression coefficient	t value	*p*-value
**Constant**	70.356	4.525		15.548	.000
**Place of residence**					
Urban	—				** **
Rural	−2.933	.987	−.145	−2.972	.**003**
**Stool trait**					
Type IV or above	—				** **
Type I	−7.012	1.198	−.334	−5.851	.**000**
Type II	−11.288	1.927	−.373	−5.857	.**000**
Type III	−10.628	2.756	−.201	−3.856	.**000**

Bold values: *p*<0.05.

*R* = 81.7, *R*^2 ^= 66.7, Adjusted *R*^2 ^= 64.2.

## 4. Discussion

### 4.1. Current status of caregiving needs

Totally 88.7% of the parents hoped or very much hoped to receive support, but only 20.85% said they had received help from medical personnel, indicating the existence of unmet care needs, and parents urgently needed the support from medical personnel. Research by Thompson (25) also showed that parents of children with FC had unmet needs for information, validation, and support, which is consistent with our study. In all dimensions, the score of information needs was the highest, mainly including disease-related knowledge and medication management. The reason may be that although clinicians can easily obtain reliable and high-quality evidence about childhood constipation, parents lack the same access, thus health care providers have become a reliable source of information. Besides, a study has pointed out that parents typically seek help from the healthcare system after living with their child's symptoms for months or years (26). Even after receiving an FC diagnosis, there is persistent parental ambiguity about the cause of the child's symptoms (27). Therefore, professional guidance is urgently needed after obtaining a diagnosis of the disease. In recent years, social media has become a source of health information for parents (28). However, confusion and misunderstanding among parents appeared with the application of professional vocabulary and the complexity of information (29, 30), in consequence, healthcare professionals were asked to verify the information on social media (31). Usually, Parents regard meeting their support and information needs as a turning point to rekindle hope and enhance confidence (23). Then, exploring multiple channels to meet the information needs of family members will never be outdated.

Our research demonstrated that psychological needs were lower than other needs, but previous studies (23, 32) noted that shame, embarrassment, and guilt were common obstacles faced by parents when providing medical care to children with FC. Probably due to the popularity of modern health perceptions, parents are more concerned about disease progression, symptom control, and complication prevention. In addition, the lack of awareness of the relationship between illness and negative emotions may be another reason for parents' neglect of psychological needs. Except for the parents themselves, a previous study (33) found that parents' experiences with FC children were often misunderstood by healthcare professionals. The study of Borowitz (34) also showed the same results. The low psychological needs of parents and the underestimation of the psychological needs of medical staff are both reminding us that this is a phenomenon worth thinking about. On the one hand, the positive emotions of parents are very important for the management of constipation in children (35). On the other hand, double neglect of psychological problems is bad for communication between parents and healthcare providers. This reminds us that it is worthwhile to devote attention to their psychological state and emotional changes, whether parents are aware of it or not. We, therefore, recommend the use of tools to monitor parents' psychological state, if necessary, to provide professional psychological guidance to those who need it.

### 4.2. Factors associated with caregiving needs

Our study demonstrated that the place of residence was an important influencing factor of parental care needs, that was, the score of living in rural areas was lower than that of children living in urban areas (*p *= 0.003), which is similar to the results of other studies (19). In our study, 46.4% of the respondents lived in rural areas. A previous study has shown that the health literacy level of urban residents was higher than that of rural residents (36). Most of the parents from rural areas lack basic awareness of the disease or had difficulty understanding the outcome of the disease. In other words, rural residents have insufficient knowledge of the disease and lack the initiative to acquire disease-related content.

In addition, quality medical resources are more accessible in cities, but not in rural areas making it impossible to form a reasonable medical orientation, resulting in a lag in the development of medical security and accessibility in rural areas (37). There are still some problems in the equalization of basic medical and health services in urban and rural areas in China, such as insufficient aggregate and unbalanced supply structure. Therefore, the public health resources obtained by rural residents are relatively limited compared to urban residents (38). Accordingly, parents' confidence in local medical care may be affected by those reasons. Simultaneously, rural residents often need to seek medical treatment in different places, so they were less dependent on the medical treatment place, which affects their caregiving needs. Hence, when we carry out nursing health education and nursing intervention, we should take the patient's residence as an important factor to formulate a scientific and standardized individual family nursing model, thereby improving treatment compliance, and improving the clinical symptoms of children.

In addition to the place of residence, children's stool traits were also the factors affecting the parents' nursing needs (*p* < 0.001), that is, the stool traits were assessed according to the Bristol stool scale, and the harder the stool trait, the higher the parental needs score. This is consistent with the findings of the same type of research (39, 40). A previous study showed that large and hard stool was the most frequent symptom among children with functional constipation, hard stool consistency was found in about 93.7% of cases (41). In the diagnosis of FC and identification of early warning symptoms (42), parental perception of constipation depends primarily on stool traits and frequency of defecation, which is also the most common reason for seeking medical attention. This suggested that parents were more likely to ignore other symptoms, leading to delays in seeking medical treatment. This was also proved by a study in Sri Lanka which showed that only 24% of young children with FC were seen by a medical doctor (43). On the other hand, ignorance of signs and symptoms indicated insufficient awareness of the disease and could affect treatment adherence (33). To achieve consensus on child care, new policies should be mindful of the long period required to guarantee interaction and counseling between parents and healthcare professionals. In addition, parents should be assisted to identify the symptoms and signs associated with the disease and improve their understanding of the disease during consultations and care planning.

In summary, our findings highlighted the caregiving needs of parents caring for a child with FC. The strength of this study lies in the large sample of parents of children with constipation and the large response rate we achieved due to patient pre-survey explanatory instructions and the preparation of small gifts. In addition, our study quantified the different needs of parents in various domains, giving us an intuitive understanding of the needs of parents of FC children. In future clinical practice, clinicians should consider the needs of parents of children, especially the information needs, when communicating with their families about constipation, and conduct personalized communication according to the specific situation of children and parents. According to the information provided by the questionnaire, it is very helpful to organize health education lectures regularly to meet the nursing needs of parents. Create a communication platform to provide opportunities for parents to share information and exchange experiences. The construction of an information-oriented hospital also provides a new communication method for parents who live in rural areas. Medical staff should pay attention to publicizing and using the information facilities of the hospital.

### 4.3. Limitations

Several limitations of this study should be taken into consideration. Firstly, A cross-sectional design does not explain causality. Secondly, the results are not necessarily representative because of convenience sampling. Thirdly, the study sample was drawn from only one hospital, which may limit the generalization of the findings. A multicenter study with a wider geographic range of selection is recommended. Future studies should employ longitudinal research methods to observe dynamic changes in caregiving needs.

## Data Availability

The raw data supporting the conclusions of this article will be made available by the authors, without undue reservation.
